# Theta band high definition transcranial alternating current stimulation, but not transcranial direct current stimulation, improves associative memory performance

**DOI:** 10.1038/s41598-019-44680-8

**Published:** 2019-06-12

**Authors:** Stefan Lang, Liu Shi Gan, Tazrina Alrazi, Oury Monchi

**Affiliations:** 1 Hotchkiss Brain Institute, Cumming School of Medicine, Calgary, AB Canada; 2 Department of Clinical Neurosciences, University of Calgary, Calgary, AB Canada; 3 Non-Invasive Neurostimulation Network, University of Calgary, Calgary, AB Canada; 4 Department of Radiology, University of Calgary, Calgary, AB Canada

**Keywords:** Cortex, Cognitive neuroscience

## Abstract

Associative memory (AM) deficits are common in neurodegenerative disease and novel therapies aimed at improving these faculties are needed. Theta band oscillations within AM networks have been shown to be important for successful memory encoding and modulating these rhythms represents a promising strategy for cognitive enhancement. Transcranial alternating current stimulation (TACS) has been hypothesized to entrain and increase power of endogenous brain rhythms. For this reason, we hypothesized that focal delivery of theta band electrical current, using high-definition TACS, would result in improved AM performance compared to sham stimulation or transcranial direct current stimulation (TDCS). In this pilot study, 60 healthy subjects were randomized to receive high definition TACS, high definition TDCS, or sham stimulation delivered to the right fusiform cortex during encoding of visual associations. Consistent with our hypothesis, improved AM performance was observed in the TACS group, while TDCS had no effect. However, TACS also resulted in improved correct rejection of never seen items, reduced false memory, and reduced forgetting, suggesting the effect may not be specific for AM processes. Overall, this work informs strategies for improving associative memory and suggests alternating current is more effective than direct current stimulation in some contexts.

## Introduction

Memory for the relationship or association between two items (associative memory; AM) is critical for normal everyday functioning. In this type of memory, individual experiences, objects, or words are linked together directly, or through spatial, temporal, or other kinds of relationships. Associative memory is central to episodic memory abilities^1,2^. Examples include remembering a person’s name or remembering the details of where you met a specific person. Associative memory can be considered verbal, such as when remembering a name which is associated with a face, or visual, such as when remembering the link between two visual objects. Difficulties with associative memory can result in disabling cognitive impairment, like those most readily seen in Alzheimer’s Disease^3^. Encoding of associative information occurs in distributed brain networks involving the left inferior frontal cortex, bilateral fusiform cortex, bilateral medial temporal lobe, bilateral premotor cortex, and bilateral posterior parietal cortex^4–6^. The modality of information to be remembered influences the relative extent of activation within this distributed network. For example, in a large meta-analysis examining neural regions involved in the subsequent memory effect of verbal and non-verbal(visual) information, it was found that the fusiform cortex was preferentially involved in the encoding of successful visual associative memory^4^. While this region is well known for its involvement in encoding face stimuli^7^, these meta-analytical results support a more general involvement of the fusiform region in visual memory encoding. Indeed, the inferior occipital-temporal region, of which the fusiform cortex is a part of, has long been hypothesized to have critical mnemonic functions^8^. Other research points to a role of the fusiform region in encoding of specific and general visual features^9^, and a role in the development of visual expertise^10^. Supporting a more general role in visual mnemonic functions, the fusiform region is also commonly implicated in disorders of cognition such as Alzheimer’s Disease^11^ and Parkinson’s Disease Dementia^12–14^. Along with the structure of associative brain networks, it is important to consider the neural dynamics which contribute to successful memory. In particular, theta band oscillations within the associative memory network are thought to play a critical role in the encoding of memories^15–19^, with a lateralization of these frequency effects seen in the right temporal lobe^16^.

Transcranial electrical current stimulation (TES) delivers electrical activity into the brain via electrodes attached to the scalp^20^, and has recently been used to modulate cognitive performance in a wide-variety of paradigms^21^. The electrical current is typically delivered in a relatively non-focal manner using a pair of electrodes placed on the scalp (1 × 1 electrode configuration). However, multi-electrode configurations (refered to as High Definition; HD) have been shown to result in a more focal electrical field distribution^22,23^. Typical HD electrode set-ups utilize a 4 × 1 ring configuration, where one center electrode is surrounded by 4 return electrodes. More recent multi-channel devices (MxN HD) with individual control of current intensity at each electrode allow for unique combinations of electrode locations combined with current optimization algorithms to more focally target brain regions^24^.

Modulation of associative memory with TES has been mostly limited to application of direct current (TDCS) paradigms during verbal associative memory tasks. Stimulation using 1 × 1 TDCS applied to the left dorsolateral prefrontal cortex (DLPFC)^25,26^ or left inferior frontal gyrus (IFG)^27^ during encoding of a face-name task improved memory performance. These positive results are contrasted by the findings of Gaynor *et al*.^28^ and Leach *et al*.^29^. Gaynor *et al*. found impaired memory performance when 1 × 1 anodal TDCS was applied to the left DLPFC during the encoding of semantically unrelated word pairs. Leach *et al*. applied 1 × 1 TDCS to the inferior frontal gyrus in older adults during the encoding of a face-name task and found increased false memory. While these studies show promise, their inconsistencies suggest new avenues should be explored. Indeed, a recent meta-analysis assessing the effect of TDCS on episodic memory found inconsistent and small effects. Still, the studies that used recall tasks and longer duration of stimulation showed enhancing effects of anodal tDCS^30^. Further, TDCS studies that have targeted posterior brain regions show promise for modulating both item^31–33^ and associative memory^34–36^.

In contrast to TDCS, an alternative form of TES involves the delivery of an alternating current, typically with a sinusoidal waveform.Transcranial alternating current stimulation (TACS) delivers oscillatory electrical activity into the brain via the same electrode set-up utilized in TDCS^37^. At the cellular level, it is thought that TACS sinusoidally alters the transmembrane potential, an effect which is magnified through synaptically connected neurons^38^. This gives rise to what is considered the primary mechanism, which is an amplification and entrainment of endogenous neuronal oscillations^39–42^. TACS can also have widespread effects on neuronal networks^41,43,44^ and may induce after-effects lasting up to 70 minutes if delivered for prolonged periods of time^45^. Theta TACS has previously been used to improve reaction times during performance of a visual memory task, though this study did not investigate whether memory performance (i.e number of correctly identified items) was modulated^46^. In a direct comparison of TDCS vs TACS on working memory performance, it was observed that TACS improved reaction time for hits compared to TDCS^47^. This offers some evidence that TACS might be more efficacaous in improving cognitive abilities as compared to TDCS.

Given the conflicting results obtained with TDCS, the importance of the fusiform region and theta oscillations in successful visual memory, and previous research suggesting TACS can modulate memory^46^ and may be more efficacaous then TDCS^47^, the present study utilized a strategy aimed at amplifying endogenous theta power in the fusiform region during memory encoding with MxN HD-TACS. Stimulation of the fusiform region has been shown to modulate face perception^48^ and facial working memory^49^, but has not been applied in an associative memory paradigm. We hypothesized that HD-TACS would improve visual associative memory performance compared to TDCS and sham stimulation. We included an active control group, applying anodal HD-TDCS, to investigate the necessity of the theta rhythm. To our knowledge, this study is the first to directly compare the effects of HD-TACS to those of HD-TDCS applied to the visual associative cortex on the performance of an associative memory task. This may guide future clinical therapies using non-invasive brain stimulation for memory enhancement.

## Methods

### Design

This is a single blind, between group, randomized, and sham controlled pilot trial assessing the difference between theta (6 Hz) HD-TACS, anodal HD-TDCS, and sham stimulation on visual associative memory performance.

### Subjects

**Figure 1:**
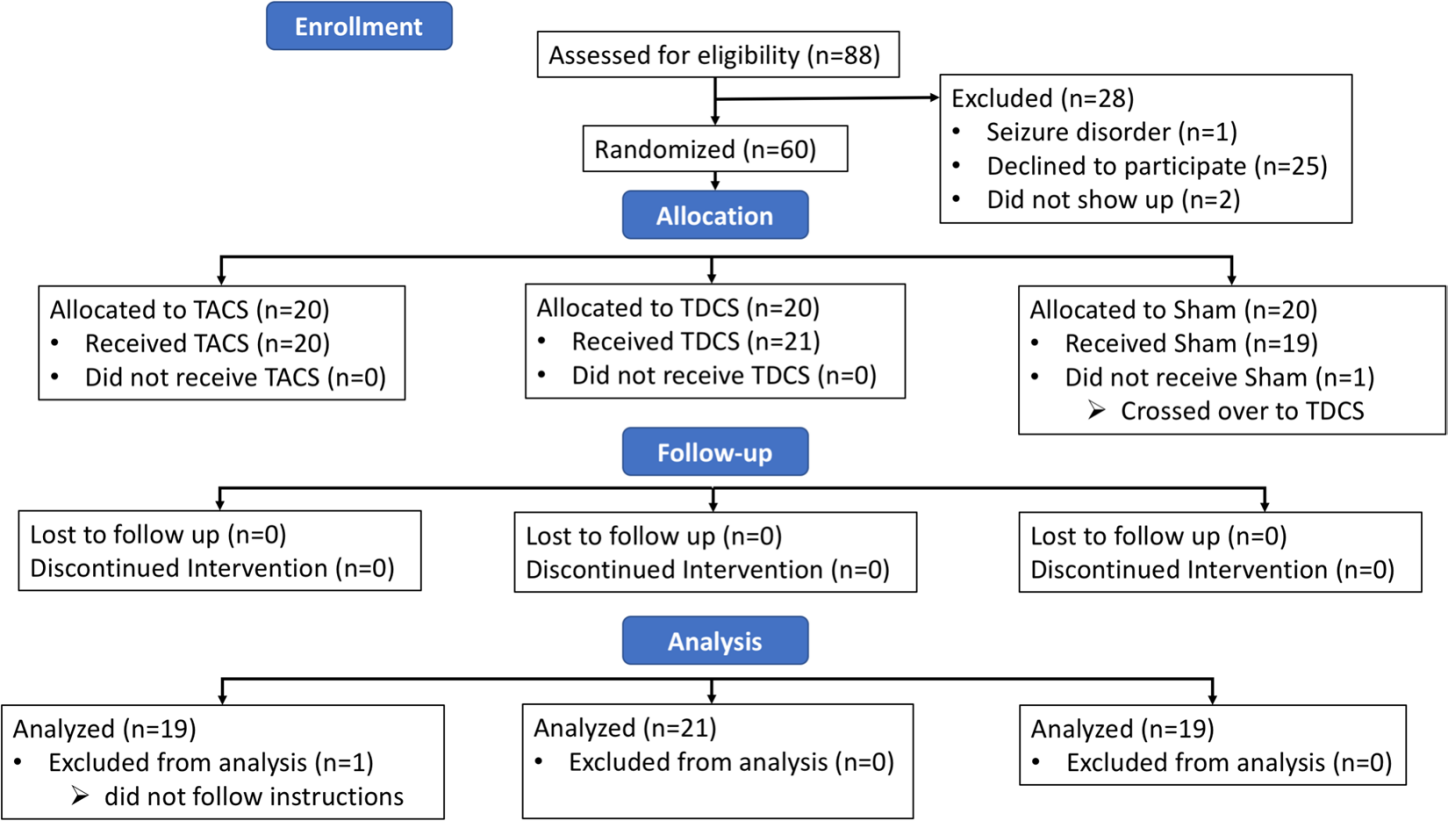
Trial Overview.

60 healthy young adults (18–45) were recruited from the university environment and underwent informed consent to participate in this protocol, which was reviewed and approved by the University of Calgary’s Conjoint Health Research Ethics Board. All methods were carried out in accordance with institutional ethical standards and with the relevant guidelines and regulations. Inclusion criteria included age between 18 and 45, no history of neurological or psychiatric disease, no recreational drug use, and no other contraindication to transcranial electrical stimulation. Participants were screened before each experiment to ensure they met these criteria and were reimbursed $40 for their participation. All experimental sessions occurred between the hours of 0730 am and 1400 pm. Subjects were randomized into one of three groups: TACS (6 Hz), anodal TDCS, or sham stimulation. One subject was excluded for failing to follow instructions, resulting in 59 subjects (TACS = 19; TDCS = 21; Sham = 19). A permutation in randomisation occurred, with one subject initially randomized to the sham group receiving TDCS due to experimenter error. Data was analysed as treated (Fig. 1). Prior to beginning the protocol, subjects completed a survey which included sleep and fatigue ratings (Supplementary Information I). Demographic information is displayed in Table 1.

**Table 1:** Subject Demographics.

Variable	TACS (Mean +/− SD)	TDCS (Mean +/− SD)	Sham (Mean +/− SD)	H/χ^2^	p value
Age (Years)	28.4 +/− 6.9	25.3 +/− 5.6	24.9 +/− 4.7	3.5	0.17
Gender (M:F)	12:7	9:12	7:12	2.92	0.57
Education (Years)	13.8 +/− 2.5	12.4 +/− 3.2	13.1 +/− 2.3	2.63	0.27
Fatigue (Subjective Rating/10)	3.05 +/− 1.5	3.64 +/− 1.4	3.16 +/− 1.9	2.1	0.35
Stress (Subjective Rating/10)	3.0 +/− 2.1	2.9 +/− 1.8	3.16 +/− 2.1	0.07	0.97

### Memory task

**Figure 2:**
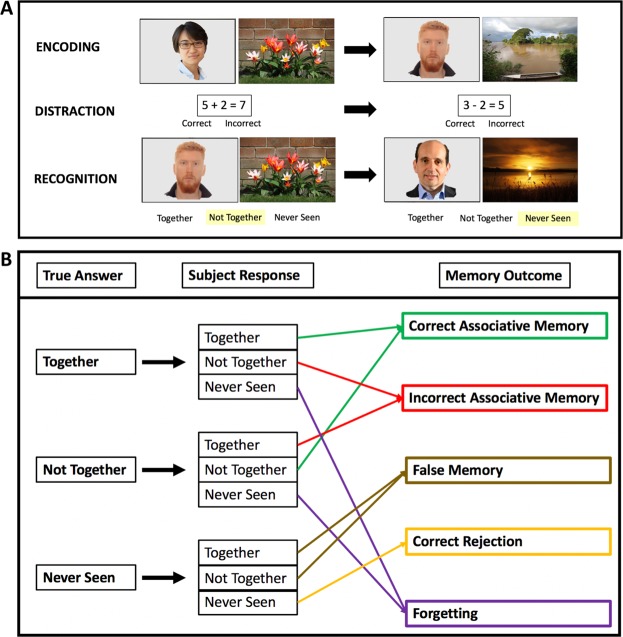
(**A**) Face and Scene Task (FAST): 27 encoding pairs are displayed sequentially, followed by a distraction task and then the recognition phase. In the recognition phase, 81 pairs are shown in random order with 1/3 consisting of ‘Together’ (Old) Pairs, 1/3 ‘Not-together’ (Lure) Pairs, and 1/3 ‘Never Seen’ (New) Pairs. (**B**) Memory Outcomes: Five memory outcomes were derived depending on the subjects response.

**Figure 3:**
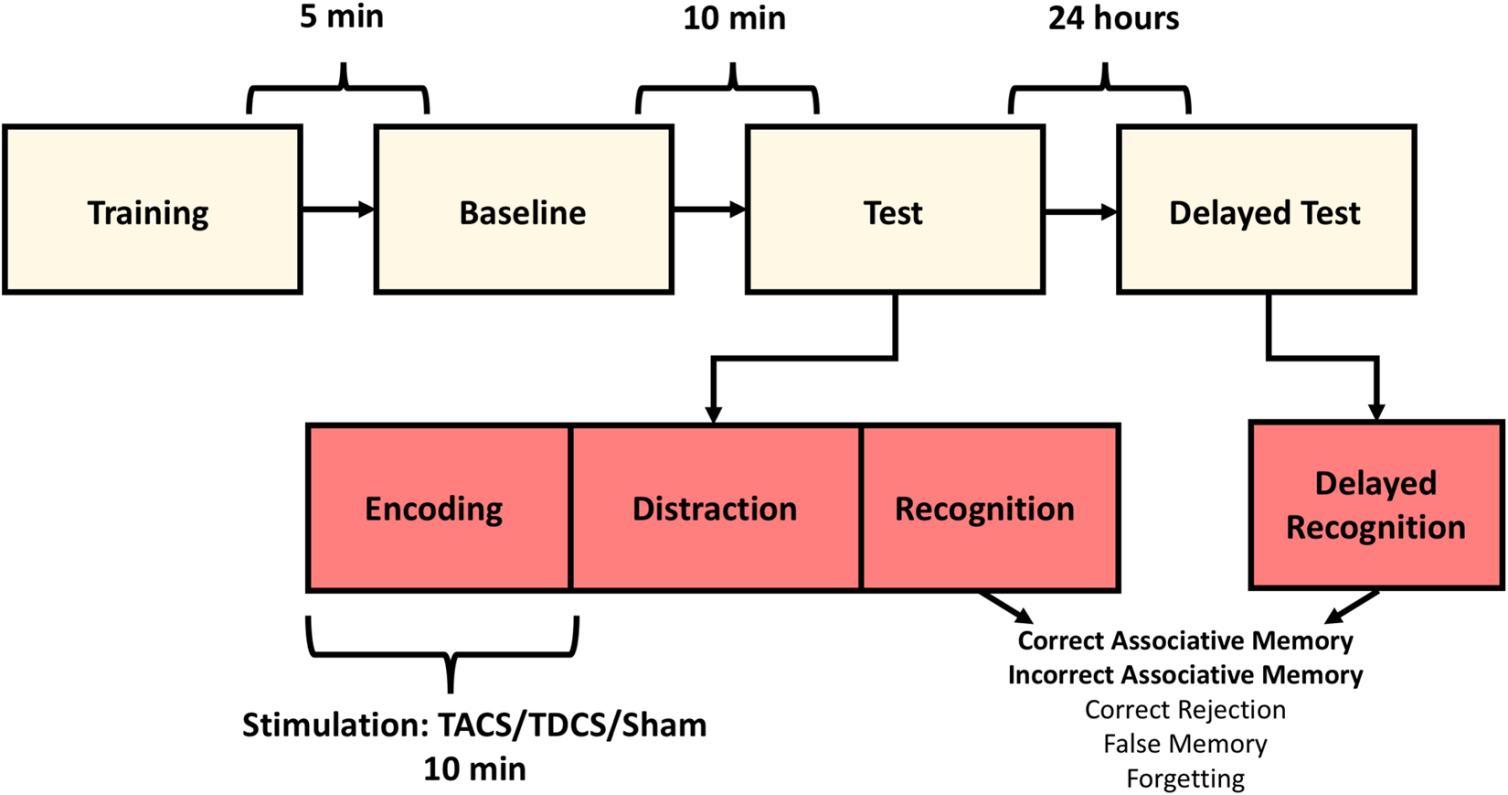
Study Protocol. Subjects undergo a brief training phase, followed by the complete FAST task to measure baseline performance. Subjects subsequently completed an alternate version of the FAST Task, with stimulation (TACS/TDCS/Sham) applied during the encoding phase. They returned 24 hours later to complete the recognition phase of the recognition test.

A visual associative memory task was designed in-house, called the Face and Scene Task (FAST). Previous studies have used similar face-scene tasks to investigate associative memory^50–52^, and have shown activity in the fusiform cortex increases for correct memory^52^ and declines with age^51^. In FAST, pictures of a face and outdoor scene are displayed side by side on a computer screen. Faces are of various ethnicities and ages, and all display a neutral expression. Scene pictures were drawn from an online public database (www.pixabay.com), while face pictures were drawn from the Park Aging Mind Laboratory Face Database^53^. Subjects were asked to remember the association between the Face and Scene, and were explicitly told their memory would be tested following the memorization/encoding phase. Picture pairs were presented for six seconds each, with a three second inter-stimulus interval. Subjects viewed 27 picture pairs during the encoding phase. This number of picture pairs was chosen based on pilot data suggesting an optimal trade-off between minimzing the ceiling effect and ensuring subjects were not responding at chance levels. Following the encoding phase, subjects completed a distraction task where they evaluated a series of simple arithmetic expressions. Twenty-five simple math expressions were presented on the screen, and subjects were instructed to identify whether they were correct or not. This distraction phase was implemented to limit recency effects while being sufficiently distinct to not engage cognitive processes involved in the FAST task^54^. Immediately following the distraction phase, the recognition phase commenced. In this phase, 27 picture pairs, consisting of the same picture pairs the subjects had memorized (called ‘together’ pairs), were randomly presented with 27 ‘lure’ pairs (consisting of a face and scene previously seen but not in the correct pairing) and 27 new pairs (consisting of a face and scene which the subjects were not exposed to during encoding). Lure pairs always consisted of both a face and scene which had previously been presented. Subjects were required to determine whether the pictures pairs were previously seen ‘together’, ‘not together’ or ‘never seen’, respectively (Fig. 2A). A maximum limit of ten seconds per picture pair was given to perform the recognition task. The primary outcomes for analysis were *Correct Associative Memory* and *Incorrect Associative Memory*. Correct Associative Memory was the proportion of responses which correctly identifies ‘together’ pairs and ‘lure’ pairs. Incorrect Associative Memory was defined as the proportion of answers which misidentifies ‘lure’ pairs as being previously seen together and ‘together’ pairs as being previously seen, but not in the same pairing. Three other memory outcomes were analyzed as secondary outcomes: Correct Rejection, False Memory, and Forgetting. Correct Rejection occurred when subjects correctly identified never seen picture pairs, False Memory occurred when subjects identified never seen picture pairs as being previously seen (together or not together), and Forgetting occurred when subjects answered ‘never seen’ to pictures which had previously been seen (Fig. 2B). Each subject performed two different versions of the task, once without stimulation (baseline performance) and once with stimulation during encoding (stimulation performance). The versions had the exact same format, but each had a unique set of picture pairs. The order of the two versions were counter-balanced between participants, though stimulation always occurred during the second presentation to mitigate influence of stimulation after-effects^45^. Primary outcomes were compared between groups on this second administration of the task. To familiarise subjects with the task, a training phase was administered prior to the baseline performance, in which a separate, abbreviated version of the task was performed with five encoding pairs and 15 recognition pairs (five ‘together’, five ‘lure’, and five never seen pairs). Twenty-four hours after the intitial memory testing, subjects returned to test for any prolonged effects of stimulation. On this visit, subjects were asked to perform the exact same recognition tests (without encoding) that they had performed the day prior. The same memory outcomes were calculated based on their performance. The study protocol is represented in Fig. 3.

### Stimulation

**Figure 4:**
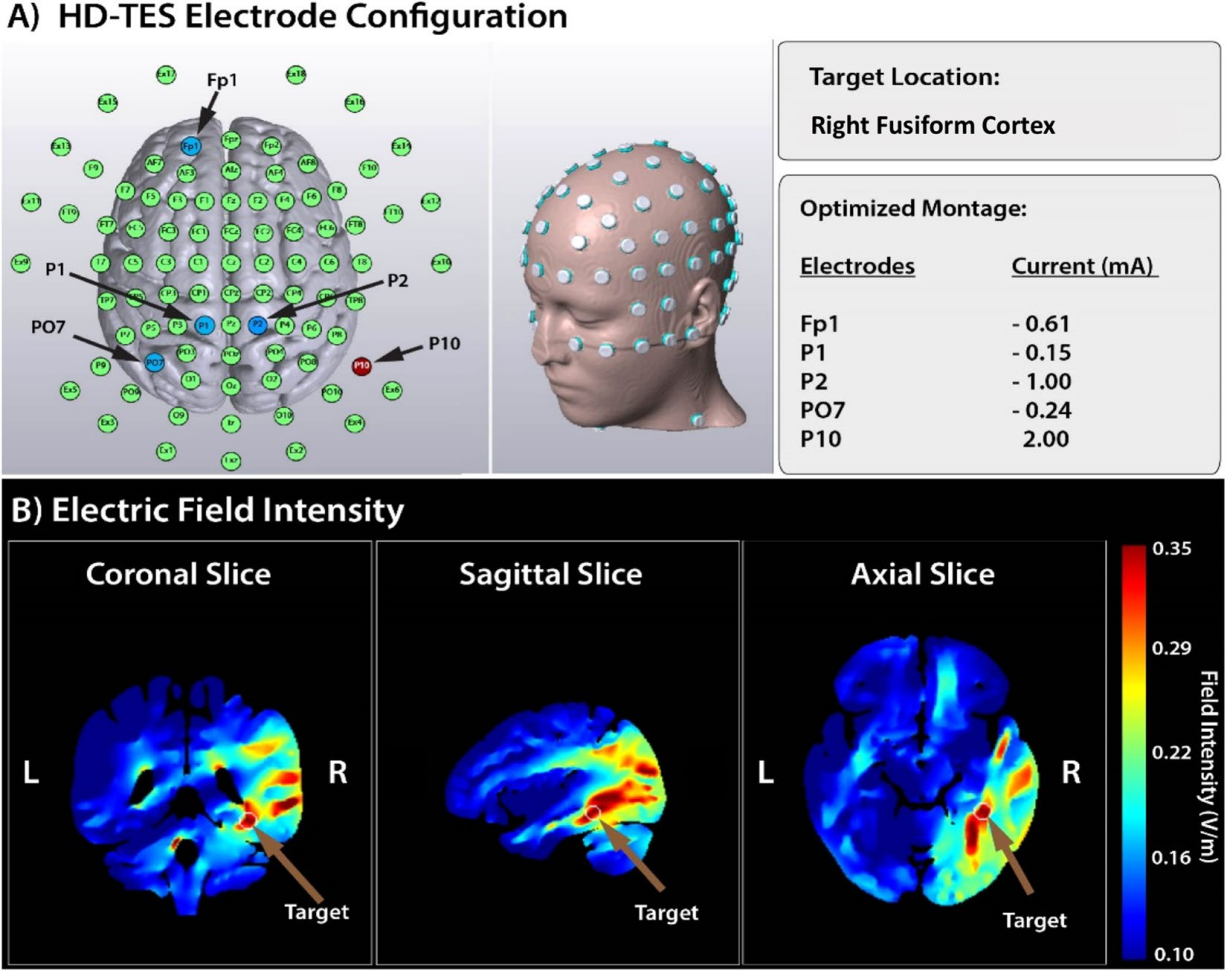
High definition Transcranial Electrical Stimulation (HD-TES). (**A**) Soterix MxN HD-TES system and the HD-Targets software were used to define the optimal electrode montage to focally stimulate the right fusiform cortex. (**B**) Finite element Modelling based on a normal adult brain template (HD-Targets software) demonstrating a high focality of electric field in the right fusiform cortex (model based on anodal direct current stimulation).

HD-TES was administered using the Soterix MxN TES device (Soterix Medical Inc., New York, USA). Electrode positions were selected using HD-Targets software (Soterix Medical Inc., New York, USA) which uses a finite-element model of a template adult brain to estimate the current distribution. This modelling software has previously shown good correspondence with other complex computational current models^55^. While modelling was performed only for TDCS, these models should also be applicable for TACS^56,57^. Stimulation sites were chosen to result in the highest focality within the right fusiform cortex. Based on this modelling, electrodes were placed at FP1, P2, P3, PO7, and P10 (Fig. 4). The same montage was used for TDCS, TACS, and sham stimulation. P10 acted as the anode, delivering 2 mA of current (peak to baseline), which was returned to the remaining electrodes based on the optimized current modelling by the HD-Targets software. The current balance at each return electrode was as follows: FP1 = −0.62 mA; P2 = −0.99 mA; P3 = −0.14 mA, and PO7 = −0.24 mA. Conductive gel (HD-GEL™, Soterix Medical Inc, New York, USA) was used to optimize conductivity and minimize impedance. While the specific imedence for each electrode was not documented, they were kept below 20kOhm. Stimulation time was set to ten minutes, with a 30 second ramp-up and ramp-down time for the active conditions. During sham stimulation, the current was ramped up to 2 mA over 30 seconds, prior to being ramped down over the next 30 seconds to 0.06 mA, where it remained for the following 9 minutes. At the end of the stimulation session, the current was again ramped up to 2 mA over 30 seconds. This procedure is commonly used to blind participants in TES studies^27,58,59^. For the first five minutes of stimulation, subjects were asked to relax and let their mind wander. For the second five minutes of stimulation, subjects performed the encoding phase of the FAST task. This delay prior to commencing the memory task was included because previous research has suggested significant effects of tDCS on cortical excitability following five minutes of stimulation^60^. To assess tolerability with each stimulation paradigm, all participants completed an adverse effect survey after the first 60 seconds of stimulation, where they rated their subjective experience of itching, burning, tingling, and discomfort on a scale of 1–5. The average of these scores constituted the overall side-effect profile (Supplementary Information II). Each subject was also asked which type of stimulation they received. This data was coded such that the answers were grouped into one of three categories: Active, Sham, or Unknown.

### Memory encoding survey

After completion of the protocol, subjects filled out an encoding strategy survey assessing subjective ratings of memory confidence and attention to the face or scene. (Supplementary Information III).

### Statistics

All data analyses were performed with MATLAB (MathWorks®, MA, USA). Normality of data was assessed with one sample Kolmogrov-Smirinov tests. To assess for our primary outcomes of stimulation induced changes in memory, and to account for the repeated measures nature of the data and the intersubject variability in performance at baseline, we utilized linear mixed effect models with a random effect of subject and fixed effects of condition (baseline and stimulation condition) and group (TACS, TDCS, and Sham). This was performed with MATLAB’s function *fitlme*, which estimates parameters of the model using maximum likelihood estimation. We were primarily interested in the interaction terms of condition and group, which gave insight into whether the dependent variable (memory scores) changed as a function of condition and group. TACS was used as the reference group given our aim of directly comparing TACS with TDCS and Sham. We used a separate model for each of our memory outcomes. Secondarily, were were interested in exploring whether any effects would persist at 24 hours, without any additional encoding. To examine this we performed similar linear mixed models for each memory outcome on the delayed follow-up. Finally, we calculated the sensitivity index (d’) as a measure of participants ability to discriminate between signal and noise^61^ (Supplementary Information IV).

Age, education, side-effect profile, memory encoding confidence, and subject ratings of focus to the face or scene showed non-normal distributions and were compared with Kruskal-Wallis tests, followed by Mann-Whitney U tests when appropriate. To test for equal proportions of gender within each group, a χ^2^ test was utilized. To formally test for blinding, we also used a χ^2^ test. We assessed for equal proportions of subjects in each treatment arm who responded with ‘Active’, ‘Sham’ or ‘Unknown’ when directly asked which type of stimulation they recieved. Significance was set at p < 0.05 for all analyses.

## Results

### Demographics

There was no significant difference between groups in age, gender, education, fatigue, or stress ratings (Table 1). None of these variables were linearly related to our primary memory outcomes (p > 0.05).

### Side-effect profile

There was no significant difference in overall stimulation side-effect profile reported by the subjects, suggesting blinding was effective. Importantly, subjects were unaware of which stimulation condition they received when directly asked (χ^2^ = 5.157, p = 0.2716). However, analysis of the individual side-effects revealed a group difference in the subjective rating of ‘tingling’ (Table 2). Post-hoc testing demonstrated this resulted from subjects receiving TACS reporting higher ratings than Sham (U = 450, p = 0.017). There was no significant difference between TACS and TDCS in the tingling rating. TACS also caused 47% of subjects to report feelings of a ‘bouncing/shaking visual field’ which consistently went away prior to commencement of the encoding phase. This effect was not reported in the TDCS or sham condition. To investigate this effect further, we examined post-stimulation correct associative memory scores between those in the TACS group who experienced the side-effect (n = 9; mean score +/− SEM = 47.60 +/− 2.00) and those that did not (n = 10; 48.33 +/− 1.28) with a Mann-Whitney U Test. No significant difference was observed (U = 89.0, p = 0.949). All subjects tolerated stimulation.

**Table 2:** Stimulation Side-Effect Profile.

Side-Effect (Subjective Rating/5)	TACS (Mean +/− SD)	TDCS (Mean +/− SD)	Sham (Mean +/− SD)	H	p value
Itching	1.52 +/− 1.4	1.14 +/− 1.4	1.68 +/− 1.3	2.38	0.30
Burning	0.53 +/− 0.8	1.09 +/− 0.9	0.74 +/− 1.0	4.4	0.11
Tingling	2.26 +/− 0.8	1.48 +/− 0.9	1.21 +/− 1.0	6.28	**0**.**04**
Discomfort	1.42 +/− 1.5	1.0 +/− 1.2	1.47 +/− 1.5	1.27	0.53
Overall	1.43 +/− 0.9	1.18 +/− 0.8	1.28 +/− 0.9	1.28	0.53

### Memory outcomes

**Figure 5:**
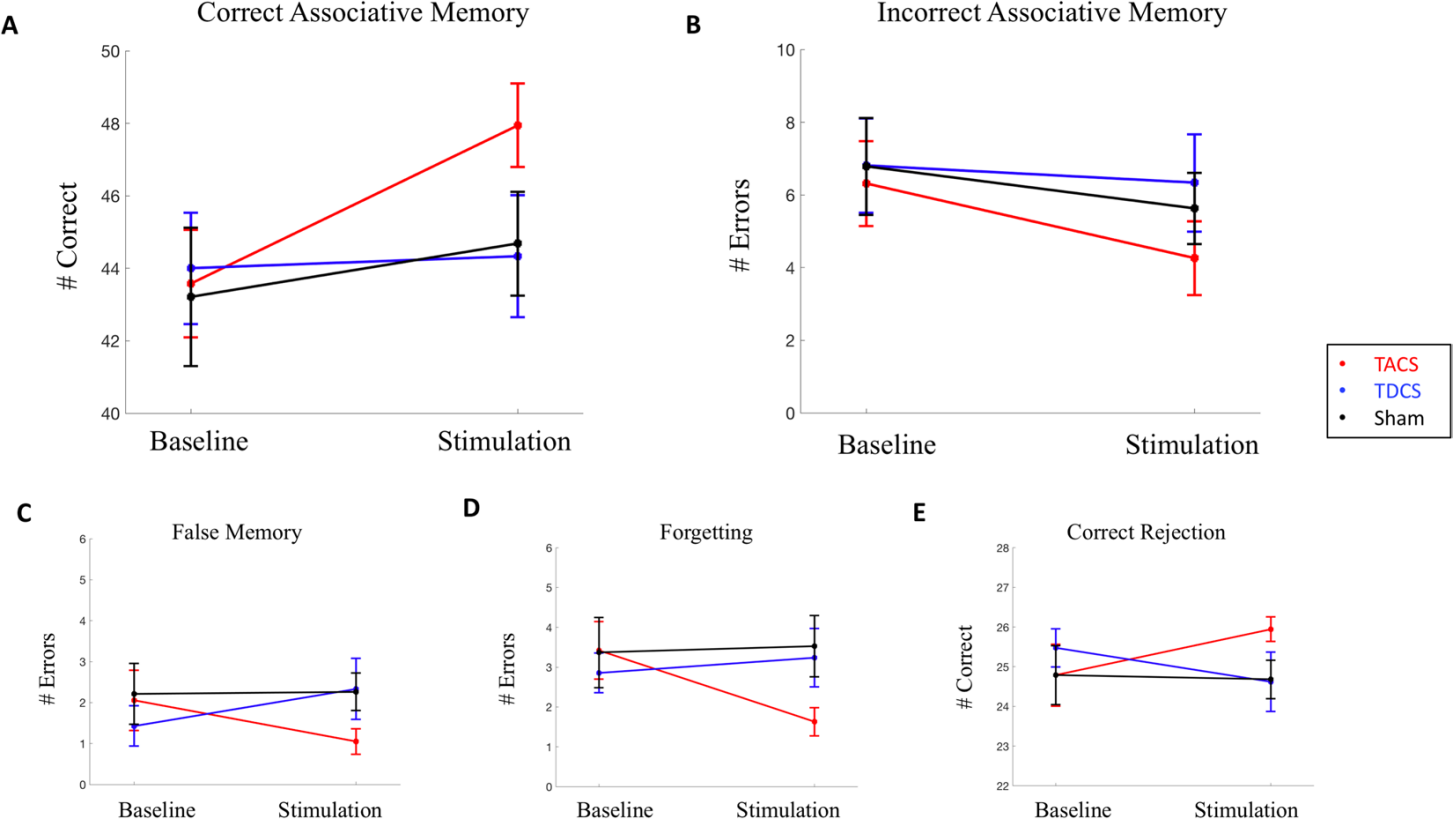
Immediate Memory Performance. TACS is the reference group. (**A**) Correct Associative Memory: Significant condition*group interaction, with TACS demonstrating improved memory performance compared to TDCS. (**B**) Incorrect Associative Memory: No difference between groups. (**C**) False Memory: Significant condition*group interaction, with TACS demonstrating less errors compared to TDCS. (**D**) Forgetting: Significant condition*group interaction, with TACS demonstrating demonstrating less errors compared to TDCS and compared to Sham. (**E**) Correct Rejection: Significant condition*group interaction, with TACS demonstrating improved rejection compared to TDCS.

**Figure 6:**
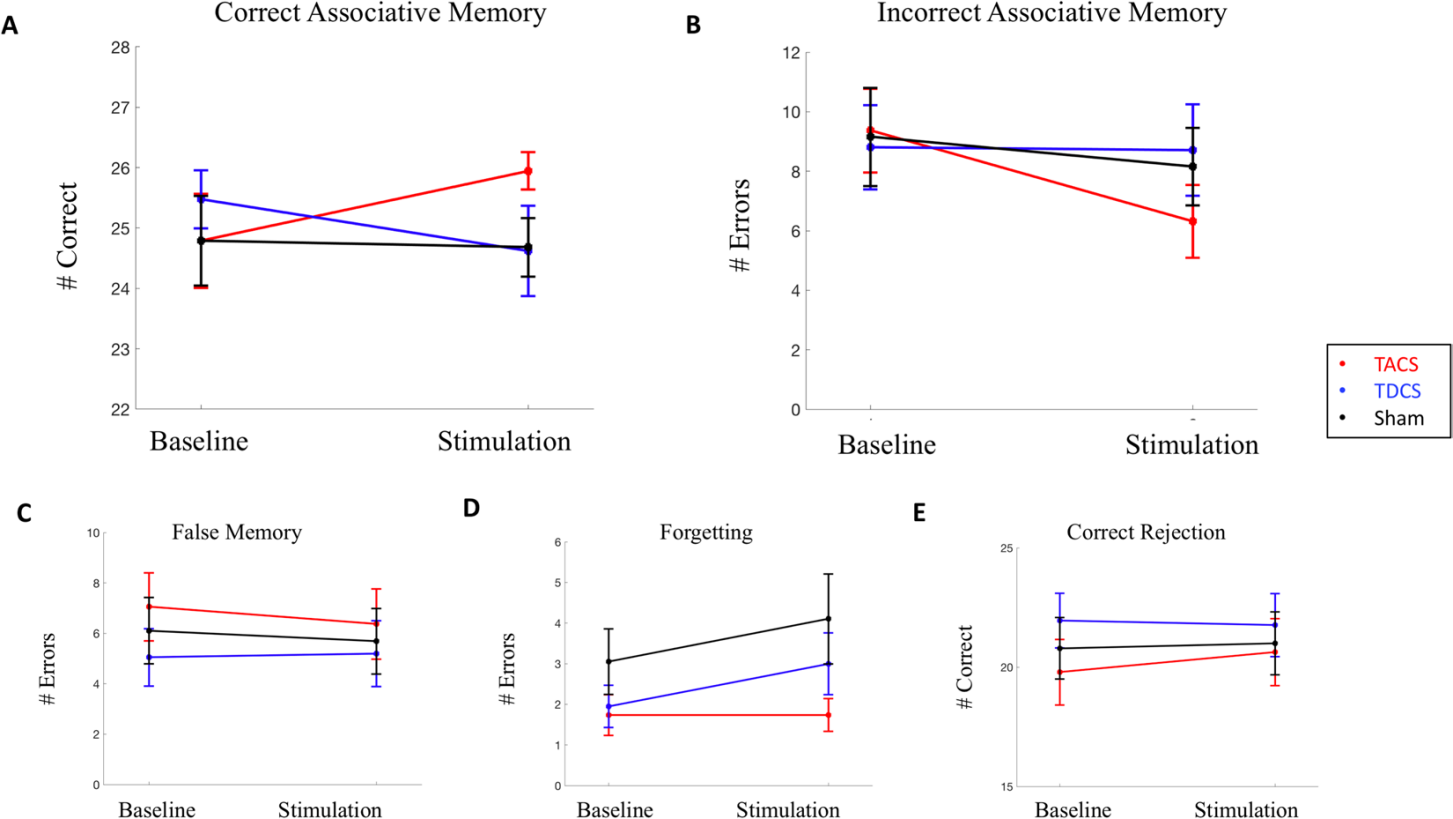
Delayed Memory Performance. TACS is the reference group. (**A**) Correct Associative Memory. (**B**) Incorrect Associative Memory. (**C**) False Memory. (**D**) Forgetting. (**E**) Correct Rejection. No statistically signficiant interactions between group and condition at 24 hours.

We performed separate linear mixed effect models for each of the memory outcomes, with TACS as the reference group (Fig. 5). The model for our primary outcome, Correct Associative Memory, demonstrated a significant main effect of condition (β = 4.368, t(112) = 3.117, p = 0.002) and a significant condition*group interaction for TDCS (β = −4.035, t(112) = −2.085, p = 0.0393). The condition*group interaction for Sham was not significant (β = −2.85, t(112) = −1.46, p = 0.147). Incorrect Associative Memory was not related to any of the fixed effects tested. The model for Correct Rejection demonstrated a significant condition*group interaction for TDCS (β = −2.015, t(112) = −2.046, p = 0.043) but not for sham ((β = −1.26, t(112) = −1.25, p = 0.213). False Memory also showed a significant condition*group interaction for TDCS (β = 1.90, t(112) = 1.99, p = 0.0493), but not Sham (β = 1.05, t(112) = 0.982, p = 0.286). The model for Forgetting demonstrated a significant main effect of condition ((β = −1.79, t(112) = −2.70, p = 0.0079), a significant condition*group interaction for TDCS (β = 2.17, t(112) = 2.38, p = 0.0191) and a significant condition*group interaction for Sham (β = 1.95, t(112) = 2.08, p = 0.0397). Details, including average memory scores for each group, can be found in Table 3. The models assessing the 24 hour delayed performance for Correct Associative Memory demonstrated a signficiant main effect of condition (β = 3.21, t(112) = 2.00, p = 0.0474), and a condition*group trend for TDCS (β = −4.02, t(112) = −1.82, p = 0.0717). There were no other significant effects for any of the additional models at the delayed time point (Fig. 6). Sensitivity index calculations were consistent with these results (Supplementary Information IV).

**Table 3:** Memory Outcomes.

Memory Outcome	Group	Condition (mean score +/− SEM)	
		Baseline	Stimulation
Correct Associative Memory	TACS	43.58 (+/−1.49)	47.95 (+/−1.15)
	TDCS	44.00 (+/−1.54)	44.33 (+/−1.69)
	Sham	43.21 (+/−1.91)	44.68 (+/−1.43)
Incorrect Associative Memory	TACS	6.32 (+/−1.16)	4.26 (+/−1.02)
	TDCS	6.81 (+/−1.30)	6.33 (+/−1.34)
	Sham	6.80 (+/−1.33)	5.63 (+/−0.97)
Correct Rejection	TACS	24.79 (+/−0.78)	25.95 (+/−0.31)
	TDCS	25.48 (+/−0.48)	24.62 (+/−0.75)
	Sham	24.79 (+/−0.74)	24.68 (+/−0.48)
False Memory	TACS	2.05 (+/−0.74)	1.05 (+/−0.31)
	TDCS	1.43 (+/−0.49)	2.33 (+/−0.75)
	Sham	2.21 (+/−0.74)	2.26 (+/−0.46)
Forgetting	TACS	3.42 (+/−0.72)	1.63 (+/−0.35)
	TDCS	2.86 (+/−0.50)	3.24 (+/−0.73)
	Sham	3.37 (+/−0.88)	3.53 (+/−0.77)

### Memory encoding strategy

There was no difference between groups in memory confidence (H(2) = 1.4, p = 0.4955) or subjective reports of attention to the face (H(2) = 3.85, p = 0.1458) or scene (H(2) = 0.62, p = 0.7323).

## Discussion

Associative memory deficits are commonly experienced during aging and are a prominent feature of many neurodegenerative diseases. Novel therapies inspired by an understanding of memory network physiology are needed. Previous attempts at improving associative memory using TES have targeted primarily the lateral frontal (DLPFC/IFG) cortex using direct current stimulation with conflicting results. The lateral frontal cortex is involved in a broad range of executive and high-level cognitive processes^62^ and may not be the ideal target for modulating associative memory directly. Attempts to modulate episodic memory have shown inconsistencies, though there may be a moderator effect of task type (recall), stimulation duration, and parietal location^30^. We have chosen to target the fusiform region due to fMRI studies suggesting a critical role in the encoding of successful visual associative memory^4^. Six Hz alternating current was chosen based on the well-known role of the theta rhythm in memory performance^15–19^, while the right side was selected based on previous literature specifically implicating right temporal theta activity in successful memory encoding^16^. One previous study targeted the right fusiform cortex with 1 × 1 anodal TDCS during a working memory task involving facial recognition^49^. In that study, stimulation enhanced demanding face working memory performance, suggesting that non-invasive brain stimulation of the fusiform cortex was possible. While the role of the fusiform cortex in face^7^ and object^63^ perception/encoding is well appreciated, this region (and more generally the inferior temporal-occipital cortex) has long been suspected of having critical mnemonic functions^8^, and has been consistently implicated in the successful encoding of visual associations^4^. This is consistent with our finding of improved visual associative memory performance. However, it is possible that the effects of stimulating this region specifically improved the encoding of faces. This possibility is supported by the observation of improved correct rejection, which is a memory process relient on item memory. We have also observed reduced false memory and reduced forgetting, suggesting a more general memory or perceptual process may have been modulated. We cannot refute this possibility with our data set, and alternative tasks will need to be utilized in the future to conclusively demonstrate an effect specific to associative memory. Despite this, the data suggest that oscillatory activity is critical for the beneficial memory results, which is consistent with the communication through coherence hypothesis^64–67^. According to this hypothesis, effective communication and information processing in brain networks is mediated through coherent oscillations between neuronal groups within those networks. Transcranial alternating current stimulation, through its hypothesised mechanism of amplifying power and entraining endogenous brain rhythms, may improve communication and information processing in neural networks. Consequently, it may be hypothesized that when TACS, at an appropriate rhythm, is targeted towards a node of a cognitive network (as done in this study), improved neural communication within the network, and ultimately improved cognitive abilities, may be expected. Our results are consistent with this reasoning.

In addition, this is the first study to directly compare HD-TACS and HD-TDCS using a MxN electrode configuration. The results suggest that TACS may be more effective than TDCS in some contexts, likely due to the reasons proposed above. Anodal TDCS had no effect on memory performance, consistent with the findings of a recent systematic review^30^. However, in that study longer duration of stimulation or the use of a recall (vs a recognition task here) were shown to moderate the effect size of TDCS stimulation, suggesting that TDCS may still be efficacious in some circumstances. Perhaps due to our relatively small smaple size, we did not observe a difference between TACS and sham stimulation on our primary outcome. However, we observed consistent trends in the same direction as TACS vs TDCS for all the memory outcomes. Furthermore, we did observe a statistical difference between TACS and sham on forgetting, supporting the claim that TACS improves memory performance.

By using the current modelling within HD-Targets software, we chose a unique electrode montage which maximizes the focality of current within the right fusiform region. Compared to traditional 1 × 1 TES, this minimizes the chances of stimulating confounding brain structures. Furthermore, previous literature targeting the dorsal anterior cingulate^68^, and recent computational modelling work, suggests that targeting deep cortical brain structures is possible with unique electrode configurations such as that utilized here^69^. The amplitude used in our TACS group is higher than that used most frequently in the TACS literature^34^. The optimal amplitude for TACS is not known, and given the lack of literature directly comparing TACS to TDCS, it is unclear which amplitudes are most comparable between the modalities. In the only other study to our knowledge performing a direct comparisons between TACS and TDCS, the TACS amplitude was chosen to be 2 mA peak to peak, resulting in a maximum amplitude which is half that of a 2 mA TDCS configuration. Nonetheless, these authors also report a beneficial effect of TACS, suggesting that perhaps lower amplitudes may also be efficaous^47^.

Several limitations must be considered when interpreting these results. Firstly, a post-hoc power analysis suggested this study was underpowered. Using the online toolbox GLIMMPSE^70^, the observed power of this study was demonstrated to be 0.493. Next, firm conclusions about the anatomical or frequency specificity of the observed effect cannot be made from this trial as no alternative region or frequency was tested. Indeed, while the computational modelling suggested the maximal intensity was focused in the fusiform cortex, it also suggested other regions of the temporal and temporal-occipital lobe received similar current intensities (Fig. 4). Alternatively, there is a possibility our unique electrode configuration resulted in the simulatenous modulation of frontal, parietal, and inferior temporal regions. This interpretation, while less likely, would suggest that the behavioural effects arose through the modulation of distributed brain regions at a theta rhythm. Therefore, we cannot conclude with certainty which region is responsible for the observed effects.

Despite the overall side-effect profile being equal between groups, post-hoc analysis suggested the TACS group experienced a higher level of a ‘tingling’ sensation compared to sham. This group also experienced, in 47% of cases, a visual side effect which to our knowledge has not been reported in the literature. This was consistently described as a ‘bouncing’ or ‘shaking’ visual field and went away prior to the commencement of the task. We cannot rule out a level of heightened attention resulting from these differences. However, we believe this is highly unlikely because no difference in correct associative memory performance was observed between those who experienced the visual side effect and those that did not. Further, there was no significant difference in side-effect profile between TACS and TDCS. Next, the extension of these results to aging and diseased populations should be considered speculative, as these biological factors can influence the result of transcranial electrical stimulation on cognitive performance^25^. Finally, we cannot rule out entrainment of brain activity resulting from peripheral nerve stimulation, though this limitation exists for most non-invasive brain stimulation research.

In summary, we have demonstrated that HD-TACS, delivered in a focal manner to the right fusiform region at a theta frequency during the encoding of visual associations, resulted in improved associative memory performance. By directly comparing HD-TACS to HD-TDCS, we find that the alternating current is crucial in the beneficial results observed. The effect occurred after a single 10-minute application of stimulation and a trend towards group differences was observed when memory was re-tested at 24 hours. This provides preliminary support for the use of high definition transcranial alternating current stimulation paradigms for memory enhancement, while also providing evidence for the role of visual association cortex in associative memory. Further research, investigating the effect of this paradigm in aging and diseased populations, testing the effect of different frequencies and anatomical locations, using longer or multiple stimulation sessions, and testing different associative memory paradigms, will be required.

## Supplementary information

Supplementary Information

## Data Availability

The data that support the finding of this manuscript are available from the corresponding author, upon reasonable request.
