# Supplementary material for: Theta band high definition transcranial alternating current stimulation, but not transcranial direct current stimulation, improves associative memory performance
Source: Sci Rep. 2019 Jun 12;9:8562. doi: 10.1038/s41598-019-44680-8 (PMC6561937; doi:10.1038/s41598-019-44680-8)
Supplement: Supplementary file 1 — Supplementary Information [file 41598_2019_44680_MOESM1_ESM.pdf]

Supplementary Information

Theta band high definition transcranial alternating current stimulation, but not transcranial direct current stimulation, improves associative memory performance

Lang, Stefan[a,b,c]., Gan, Liu Shi[a,c]., Alrazi, Tazrina[a,b]., Monchi, Oury*[a,b,c,d].

a Cumming School of Medicine, Hotchkiss Brain Institute, Calgary, AB, CA
b Department of Clinical Neurosciences, University of Calgary, Calgary, AB, CA
c Non-Invasive Neurostimulation Network, University of Calgary, Calgary AB, CA
d Department of Radiology, University of Calgary, Calgary AB, CA

I: Screening Questionnaire
II: Adverse Effect Questionnaire
II: Encoding Strategy Survey
IV: Sensitivity Index calculation

UNIVERSITY OF CALGARY
CUMMING SCHOOL OF MEDICINE

TDCS Screening Questionnaire

Participant ID #: ____

If you agree to take part in this study, please answer the following questions. The information you provide is for screening purposes only and will be kept completely confidential.

Gender: MALE FEMALE OTHER

Dominant Hand: RIGHT LEFT BOTH

Fluent English: YES NO

Age: __________ Years of Education: ___________

Screening Questions	YES	NO	Details
Have you ever suffered from any neurological or psychiatric conditions?			
Have you ever had a seizure?			
Does anyone in your immediate family have epilepsy?			
Do you suffer from migraines?			
Have you ever undergone neurosurgery?			
Do you have anything implanted in your skull or brain?			
Do you have anything implanted in your heart?			
Did you ever suffer from a brain injury?			
Did you ever lose consciousness?			
Do you suffer from chronic skin disorders?			
Are you currently taking any prescribed medication			
Are you currently taking any unprescribed medication?			
Do you use recreational drugs?			

Screening Questions	YES	NO	Details
Have you drunk any alcohol in the last 24 hours?			
Have you drunk any alcohol today?			
Have you had more than one cup of coffee, or other sources of caffeine today?<br>If Yes, how recent?			
Have you used recreational drugs recently?			
How many hours of sleep did you have last night?			
Have you ever had non-invasive brain stimulation (TMS/TDCS)?<br>If yes, when was this?			
Have you had recent stressful event in your life?			
How tired are you now, on a scale from 1-10			
How stressed are you now, on a scale from 1-10?			
Did you exercise today?			

I confirm that the information listed here is accurate to the best of my knowledge.

Name: ____

Signature: ___________________________ Date: ________________________________

This form has been verified by (member of the research team):

Name: ____

Signature: ___________________________ Date: ________________________________

UNIVERSITY OF CALGARY
CUMMING SCHOOL OF MEDICINE

Adverse Effect Questionnaire

Subject ID: ___________________________________
Date: ___________________________________
Age: ___________________________________

Adverse Effect	No	Yes	Severity	Relationship with Stimulation
Headache			1 2 3 4 5	1 2 3 4 5
Neck Pain			1 2 3 4 5	1 2 3 4 5
Discomfort under the electrodes			1 2 3 4 5	1 2 3 4 5
Discomfort distant from electrodes			1 2 3 4 5	1 2 3 4 5
Itching			1 2 3 4 5	1 2 3 4 5
Burning			1 2 3 4 5	1 2 3 4 5
Tingling			1 2 3 4 5	1 2 3 4 5
Skin redness			1 2 3 4 5	1 2 3 4 5
Fatigue			1 2 3 4 5	1 2 3 4 5
Nausea			1 2 3 4 5	1 2 3 4 5
Dizziness			1 2 3 4 5	1 2 3 4 5
Other Effects (please describe):			1 2 3 4 5	1 2 3 4 5

Encoding Strategy Survey

For each question, indicate how much this applies to your experience.
1 = Not at All
5 = Very Much

1. I felt I did very well with remembering the pictures

   1 2 3 4 5

2. I felt like I had no memory at all for any of the picture pairs

   1 2 3 4 5

3. I rehearsed the pictures by thinking about them after I had seen them

   1 2 3 4 5

4. I focused on basic perceptual features of the face (i.e eye color, skin color, hair color)

   1 2 3 4 5

5. I focused on basic perceptual features of the scene (i.e color)

   1 2 3 4 5

6. I created a conceptual link between the face and scene (i.e this face looks like a movie character and this movie character fits with this location)

   1 2 3 4 5

7. I created a conceptual link to the face alone (i.e this face looks like someone I recognize)

1 2 3 4 5

8. I created a conceptual link to the scene alone (i.e this place looks like a place I know)

1 2 3 4 5

9. I focused more on the face than on the scene

1 2 3 4 5

10. I focused more on the scene than on the face

1 2 3 4 5

11. I told myself a story about the face and scene (i.e this face is a person who might be spending time in this location because they are doing something here)

1 2 3 4 5

12. I associated the face with some emotional component (i.e this face appears scary, this face appears attractive)

1 2 3 4 5

13. I associated the scene with an emotional component (i.e I have been to a place like this and I enjoyed it; I have been to a place like this and I hated it).

1 2 3 4 5

It is likely you remembered some picture pairs well while others you may not have remembered well or may have completely forgotten.

For the picture pairs you remembered well, what was the thing(s) about it that made you remember it?

For those that you forgot completely, why do you think that happened?

Please insert any further strategies you employed while trying to remember the picture pairs, or any comments you have about why you remembered some pictures but forgot others.

Supplementary Information IV:

Sensitivity Index Methods & Results

Sensitivity index (d') was calculated as a measure of participants ability to discriminate between signal and noise[61]. Hits are defined as the z-score of Correct Associative Memory (signal present and subject detects signal), and False Alarms are defined as the z-score of False Memory (signal not present but subject detects signal). This was performed using the MATLAB function norminv(Hits) – norminv(FalseAlarms). Extreme values (Hits = 1 and False Alarm = 0) are transformed with n-0.5/n and 0.5/n respectively[61]. Results of this d-prime calculation are consistent with the overall findings of our study: TACS improves d' relative to TDCS (Group*condition interaction: β=-0.576, t(112)=-2.00, p=0.0475), while there remains a trend for TACS vs Sham (Group*condition interaction: β=-0.481, t(112)=-1.63, p=0.105). At the delayed assessment there was no difference between groups.

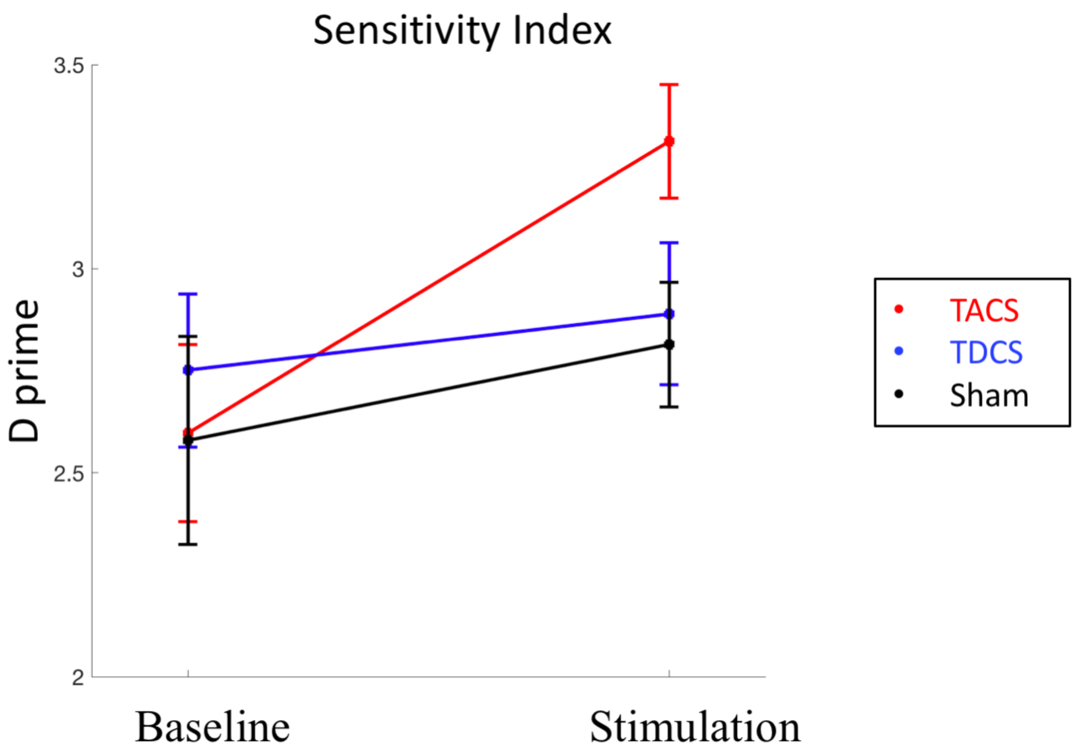

Supplementary Figure: Sensitivity Index. TACS improves d' relative to TDCS.
